# Interrelationships among common symptoms in the elderly and their effects on health-related quality of life: a cross-sectional study in rural Korea

**DOI:** 10.1186/s12955-016-0549-9

**Published:** 2016-10-13

**Authors:** Sujeong Mun, Kihyun Park, Younghwa Baek, Siwoo Lee, Jong-hyang Yoo

**Affiliations:** Mibyeong Research Center, Korea Institute of Oriental Medicine, 1672 Yuseong-daero, Yuseong-gu, Daejeon, 305-811 Republic of Korea

**Keywords:** Health-related quality of life, Elderly, Fatigue, Pain, Sleep disturbance, Indigestion, Depression

## Abstract

**Background:**

Because the world population is aging, it has become increasingly important to focus on and meet the healthcare needs of elderly individuals. This study aims to evaluate the relationships among common symptoms experienced by the elderly, including fatigue, pain, sleep disturbance, indigestion, and depression/anger/anxiety, and to assess how these symptoms affect health-related quality of life (HRQoL) in the elderly population after adjusting for sociodemographic characteristics and diagnosed diseases.

**Methods:**

In a cross-sectional study conducted in 2014 in a rural area of Korea, we extracted data on 1328 elderly individuals aged 60 years or older. Their HRQoL was assessed using the EuroQol Five-Dimension (EQ-5D) questionnaire. The pairwise associations between each symptom and the influence of the symptoms on HRQoL were measured using logistic regression and multiple regression analysis.

**Results:**

Each symptom was positively correlated with the other symptoms. The strongest association was observed between fatigue and pain (adjusted odds ratio = 8.127), and the weakest correlation was observed between sleep and indigestion (adjusted odds ratio = 2.521). Of the individuals experiencing symptoms other than sleep disturbance, those who reported comorbid symptoms tended to report higher symptom severity and a higher prevalence of symptoms persisting for ≥ 3 days compared with individuals who reported only one symptom. The number of symptoms was significantly correlated with the EQ-5D index (Spearman correlation coefficient = −0.370, *p* < 0.01) and the EQ Visual Analog Scale (EQ VAS) scores (Spearman correlation coefficient = −0.226, *p* < 0.01). Fatigue, pain, and sleep disturbance showed negative effects on all dimensions of EQ-5D. In multiple regression analysis, fatigue (*β* = −0.073, *p* < 0.01), pain (*β* = −0.140, *p* < 0.01), sleep disturbance (*β* = −0.061, *p* < 0.05), and depression/anger/anxiety (*β* = −0.065, *p* < 0.05) showed significant independent effects on the EQ-5D index when we adjusted for socioeconomic characteristics and diagnosed diseases.

**Conclusions:**

Fatigue, pain, sleep disturbance, and depression/anger/anxiety were correlated with one another, and they presented significant independent effects on the HRQoL of elderly individuals. Thus, multidisciplinary healthcare programs are required to address these common symptoms.

## Background

The world population is rapidly aging, and elderly individuals over 60 years of age accounted for 11 % of the world population in 2000, with this figure expected to double by 2050 to 22 %. This change can be attributed to longer life expectancies and decreasing fertility rates. Elderly individuals are expected to represent more than 30 % of Korea’s population by 2050 [1, 2]. Fertility rates in Korea have markedly declined over the last 50 years, and two parents in the current generation are replaced, on average, with only one child in the next generation [3]; thus, Korea has one of the lowest fertility rates among the Organization for Economic Co-operation and Development (OECD) countries. The expected increase in healthcare demands caused by this aging society presents a significant challenge for health professionals and policy makers.

A large proportion of elderly individuals experience a number of symptoms that commonly include fatigue, pain, sleep disturbance, and gastrointestinal and psychological symptoms. Moreover, these symptoms often emerge simultaneously and appear to be related to one another. Fatigue is reported to be associated with pain, depression, and sleep disturbance [4]. Similarly, pain has been found to be associated with sleep disturbances and depressed mood [5]. Additionally, people with insomnia have a higher incidence of pain and gastrointestinal problems, even when depression and anxiety levels are controlled [6, 7]. These symptoms experienced by the elderly are reported to affect their quality of life, functional dependence, and even mortality [5, 8–14].

Health-related quality of life (HRQoL) has been used as an important endpoint for understanding the life experiences of older individuals and evaluating the effects of medical treatment. Although previous studies have shown the effects of one or two symptoms on HRQoL and evaluated the associations between these symptoms, few studies of older populations have explored the interrelationships among a wider scope of prevalent symptoms and determined how these symptoms affect HRQoL. Considering the high prevalence of these symptoms, better understanding the interrelationships between the symptoms could assist in developing healthcare programs for the elderly, especially when those symptoms significantly affect HRQoL.

Accordingly, this study aims to evaluate the associations between common symptoms including fatigue, pain, sleep disturbance, and gastrointestinal and psychological symptoms to determine how they affect the presence and severity of the other comorbid symptoms. We also ascertain how they affect the HRQoL of the elderly after adjusting for socioeconomic and diagnosed diseases in the elderly.

## Methods

### Data source

This study used secondary data from a sample of 1890 residents in three rural towns (Gampo-eup, Yangnam-myeon, and Yangbuk-myeon) in Gyeongju, South Korea. The original cross-sectional research was conducted in February 2014 to evaluate the prevalence of Mibyeong (sub-health) and to investigate the relationship of a variety of clinical indices within the general population of the state of Mibyeong. The analysis included 1328 participants aged 60 years or older to assess the interrelationships among common symptoms and their effect on HRQoL in the elderly.

### Symptoms, diseases and socioeconomic characteristics

The presence of fatigue was determined by asking the following question: “Have you ever experienced fatigue in the previous month?” The respondents who answered “Yes” were asked the following questions: “Did the fatigue persist for more than 3 days?”; “Did the fatigue persist even after resting?”; “How severe was the fatigue?”; and “How severe was the inconvenience caused by the fatigue?” For the last two questions, the responses were recorded on a seven-point Likert scale ranging from one to seven. Pain, sleep problems, digestion problems, and depression/anger/anxiety issues were also assessed in the same manner. The symptoms in the questionnaire covered complaints that had been frequently reported in the general Korean population (fatigue, 70.7 %; pain, 30.8 %: sleep problems, 16.7 %; digestion problems, 18.3 %; depression, 17.3 %; anger, 18.7 %; and anxiety, 12.8 %) in a nationwide cross-sectional survey that was conducted by our research group in 2013 [15].

Twenty-seven diseases were self-reported using a questionnaire. The diseases in the questionnaire included stroke, transient ischemic attack, angina (or myocardial infarction), hypertension, dyslipidemia, pulmonary tuberculosis, thyroid disorder (other than thyroid cancer), chronic gastritis, ulcer (gastric/duodenal), diabetes, intestinal polyp, acute hepatic disease, fatty liver, chronic hepatitis (or hepatic cirrhosis), cholelithiasis (or cholecystitis), chronic bronchitis, asthma, allergy, arthritis, cystitis, cataract, glaucoma, depression, Parkinson’s disease, osteoporosis, prostatic hyperplasia, and cancer. The participants’ history of disease was determined by asking the following question: “Have you ever been diagnosed with the following diseases by a physician?” The respondents who answered “Yes” to that question were then asked whether they had received treatment for the disease and whether they were still receiving treatment.

Of the diseases assessed, three diseases (hypertension, diabetes, and dyslipidemia) were defined according to the self-report and clinical parameters. Participants were assumed to have the disease if their clinical parameters indicated the presence of the disease or if they answered that they were receiving treatment for the disease. Hypertension was considered to be present if the participant's systolic blood pressure was ≥ 140 mmHg or if his/her diastolic blood pressure was ≥ 90 mmHg. Dyslipidemia was considered to be present if one of the following criteria was met: a total cholesterol level of 240 mg/dL, low-density lipoprotein (LDL) cholesterol ≥ 160 mg/dL, high-density lipoprotein (HDL) cholesterol < 40 mg/dL, or triglycerides ≥ 200 mg/dL. Diabetes was considered to be present if the participant’s fasting glucose was ≥ 126. Additionally, the presence of obesity was determined if the participant had a body mass index (BMI) of 25 kg/m^2^ or higher based on their measured body height and weight.

The socioeconomic characteristics assessed in this study included gender, age, household income (monthly), educational level (less than elementary, elementary, middle and high school or higher), and solitary living. Information on these variables was obtained by a self-administered questionnaire.

### EQ-5D

The EQ-5D, which was developed by the EuroQol Group, is a generic measure of HRQoL. Health status is assessed across five dimensions (mobility, self-care, usual activities, pain/discomfort, and anxiety/depression) with three levels (no problems, some or moderate problems, and extreme problems). The EQ-5D has been reported to be useful for measuring HRQoL during old age [16]. The EQ-5D provides an estimate of the health summary score, which is called the EQ-5D index, by applying a formula that attaches specific values to each of the levels in each dimension. The EQ-5D index in this study was calculated using the time trade-off valuation set that pertained to the Korean population [17]. The EQ Visual Analog Scale (VAS) was used to capture the respondents’ self-rated health by asking them to rate their overall health on a vertical line with the endpoints “Best imaginable health state” (100) and “Worst imaginable health state” (0).

### Ethical considerations

This study was approved by the Korean Institute of Oriental Medicine Ethics Committee (No. I-1401/001-001-01). The study was conducted in accordance with the Declaration of Helsinki. Written informed consent was obtained from all participants before they were enrolled in the study.

### Statistical analyses

Pairwise associations between each of the symptoms were measured using a logistic regression after adjusting for gender, age, income, education, and solitary living. The differences between individuals with one symptom and individuals with two or more symptoms were assessed using Student’s *t*-test for symptom severity and a chi-square test for the prevalence of symptoms persisting ≥ 3 days. The correlations between the number of symptoms and the EQ-5D index and EQ VAS score were assessed using Spearman’s correlation coefficient. To estimate the influence of each symptom on each dimension of EQ-5D, a logistic regression analysis was conducted with the probability of reporting moderate or extreme problems in the respective EQ-5D dimensions as the dependent variables. The influence of symptoms on the EQ-5D index and the EQ VAS score was determined by multiple regression analysis. Socioeconomic factors including gender, age, income, education and solitary living were entered into the regression model as covariates with pain, sleep disturbance, indigestion, and depression/anger/anxiety. Additionally, the statuses of 28 diagnosed diseases were entered into the model using forward stepwise selection. In the regression analyses, all diseases and symptoms were considered to be binary covariates. Multicollinearity was examined using the tolerance and variance inflation factor (VIF). The results showed that the highest VIF value was 2.191 and that the lowest tolerance value was 0.456, which suggests that multicollinearity likely did not affect the analysis. A *p*-value of less than 0.05 was considered to be statistically significant. All statistical analyses were performed using SPSS 22.0 (IBM, Chicago, IL, USA).

## Results

### Sample characteristics

Of the 1328 participants, 870 (65.5 %) were women, and the mean age was 70.0 (SD = 5.9). Regarding the participants’ educational level, 37.8 % of the participants reported less than elementary school education, 40.8 % reported elementary school education, 13.5 % reported middle school education, and 7.9 % reported a high school or higher level of education. The proportion of individuals with a solitary living status was 23.9 % of all participants, including 6.1 % of the men and 33.2 % of the women. Pain was reported by 755 (56.9 %) of the participants, fatigue was reported by 728 (54.8 %) of the participants, sleep disturbance was reported by 443 (33.4 %) of the participants, depression/anger/anxiety was reported by 353 (26.6 %) of the participants, and indigestion was reported by 335 (25.2 %) of the participants. Additionally, 314 (23.6 %) participants reported that they had none of these 5 symptoms, whereas 257 (19.4 %), 274 (20.6 %), and 483 (36.4 %) participants reported one, two, and three or more symptoms, respectively. Hypertension (66.9 %) and obesity (52.0 %) were the most prevalent diseases, followed by dyslipidemia (41.8 %), arthritis (30.0 %), and diabetes (16.3 %) (Table 1). The mean EQ-5D index score was 0.814 ± 0.139, and the mean EQ VAS score was 64.3 ± 15.8.

**Table 1:** Socioeconomic characteristics and self-reported prevalence of symptoms and diseases in the study participants

Characteristics		Participants
		*N* = 1328
Mean age, years (SD)		70.0 (5.9)
Education, *n* (%)	Less than elementary school	502 (37.8)
	Elementary school	542 (40.8)
	Middle school	179 (13.5)
	High school or higher	105 (7.9)
Living alone, *n* (%)	Yes	317 (23.9)
	No	1011 (76.1)
Household income^a^, 10,000 won/month, *n* (%)	< 50	293 (23.0)
	50–99	469 (36.8)
	100–199	362 (28.4)
	≥ 200	152 (11.9)
Symptoms, *n* (%)	Fatigue	728 (54.8)
	Pain	755 (56.9)
	Sleep disturbance	443 (33.4)
	Indigestion	335 (25.2)
	Depression/anger/anxiety	353 (26.6)
Number of symptoms, *n* (%)	0	314 (23.6)
	1	257 (19.4)
	2	274 (20.6)
	3	211 (15.9)
	4	184 (13.9)
	5	88 (6.6)
Diagnosed diseases^b^, *n* (%)	Hypertension	889 (66.9)
	Obesity	690 (52.0)
	Dyslipidemia	555 (41.8)
	Arthritis	399 (30.0)
	Diabetes mellitus	217 (16.3)

^a^Fifty-two participants (3.9 %) refused to answer the question related to household income

^b^Of the collected information on twenty-eight diseases, the five most prevalent diseases are listed in the table

### Interrelationships between symptoms

The pairwise associations between each symptom are presented in Table 2. The odds ratios (ORs) were estimated after adjusting for gender, age, income, education, and solitary living. Each symptom was positively correlated with each of the others. The strongest associations were observed between fatigue and pain (adjusted OR = 8.127) and between fatigue and depression/anger/anxiety (adjusted OR = 5.157), whereas the weakest association was observed between sleep and indigestion (adjusted OR = 2.521) (Table 2).

**Table 2:** Pairwise associations between each symptom measured by ORs of the increased prevalence of one symptom in the presence of the others

	Fatigue	Pain	Sleep disturbance	Indigestion	Depression/anger/anxiety
Fatigue	-				
Pain	8.127 (6.234 to 10.596)	-			
Sleep disturbance	3.155 (2.417 to 4.119)	3.124 (2.376 to 4.107)	-		
Indigestion	3.228 (2.401 to 4.339)	3.202 (2.362 to 4.341)	2.521 (1.921 to 3.308)	-	
Depression/anger/anxiety	5.157 (3.719 to 7.151)	4.115 (2.973 to 5.696)	5.111 (3.846 to 6.793)	2.500 (1.872 to 3.338)	-

The estimated ORs by the logistic regression were adjusted for gender, age, income, education, and solitary living

**Fig. 1:**
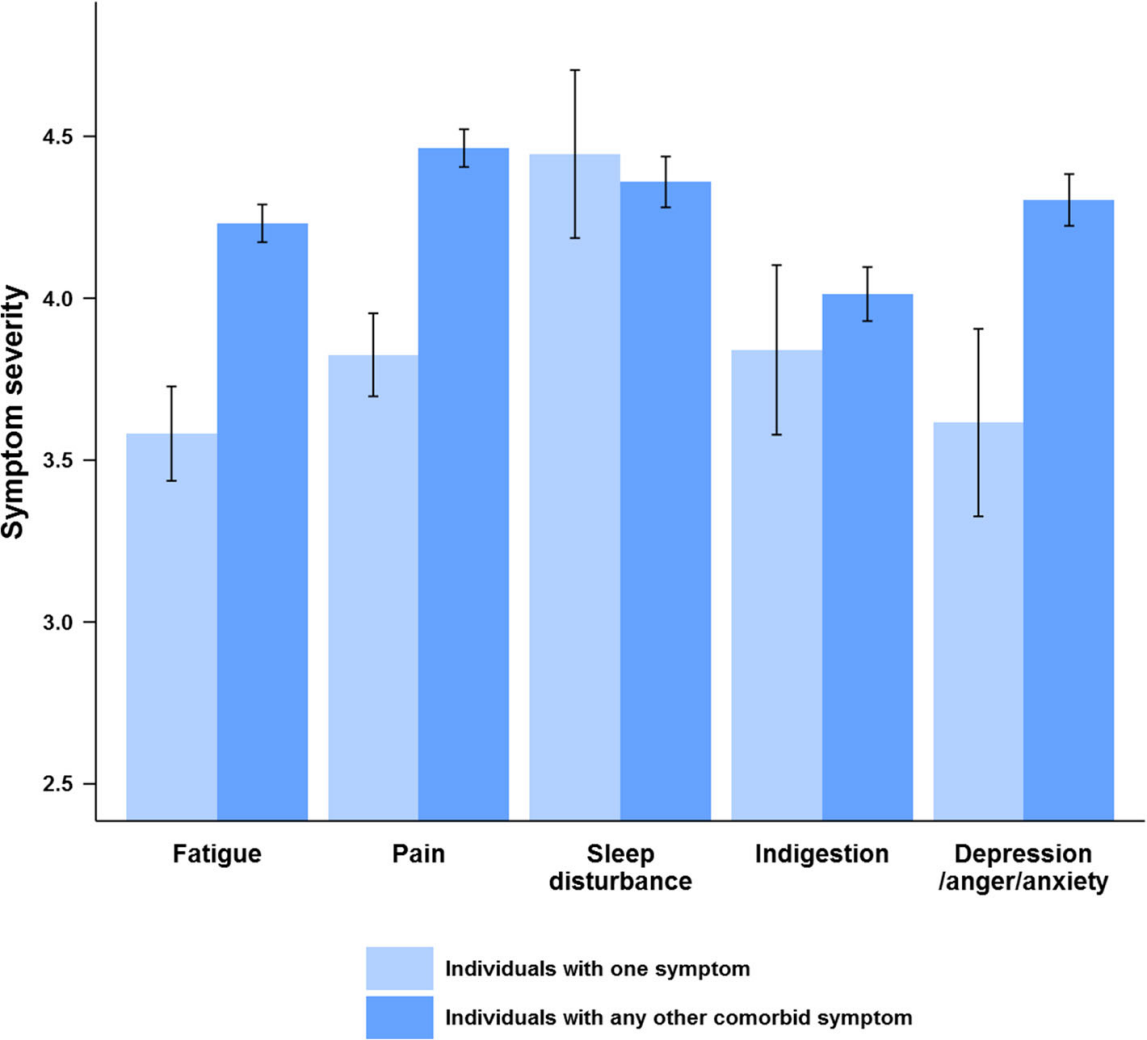
Severity of each symptom according to the presence of any other symptom. Light blue represents individuals with only one of the corresponding symptoms on the x-axis. Blue represents individuals with any other symptoms in the presence of the corresponding symptom. Symptom severity was assessed on a seven-point Likert scale ranging from one to seven

The participants who reported only one symptom (with the exception of sleep disturbance) tended to report lower symptom severity, whereas the individuals with another comorbid symptom reported higher symptom severity (Fig. 1). Similarly, the participants with only one symptom reported a lower prevalence of symptoms persisting for ≥ 3 days (fatigue: 20.9 %, pain: 49.5 %, sleep disturbance: 41.7 %, indigestion: 36.0 %, anxiety: 30.8 %), whereas the individuals with another comorbid symptom reported a higher prevalence of symptoms persisting for ≥ 3 days (fatigue: 48.0 %, pain: 58.4 %, sleep disturbance: 46.7 %, indigestion: 37.1 %, anxiety: 42.6 %). This result was statistically significant for fatigue (symptom severity, *p* < 0.01; prevalence of symptoms persisting for ≥ 3 days, *p* < 0.01), but for pain, only the difference in symptom severity was significant (*p* < 0.01). Regarding anxiety symptoms, the difference in symptom severity was not significant when equal variances were assumed (*p* = 0.10), but it was significant when equal variances were not assumed (*p* < 0.05).

### Effects of symptoms on HRQoL

**Fig. 2:**
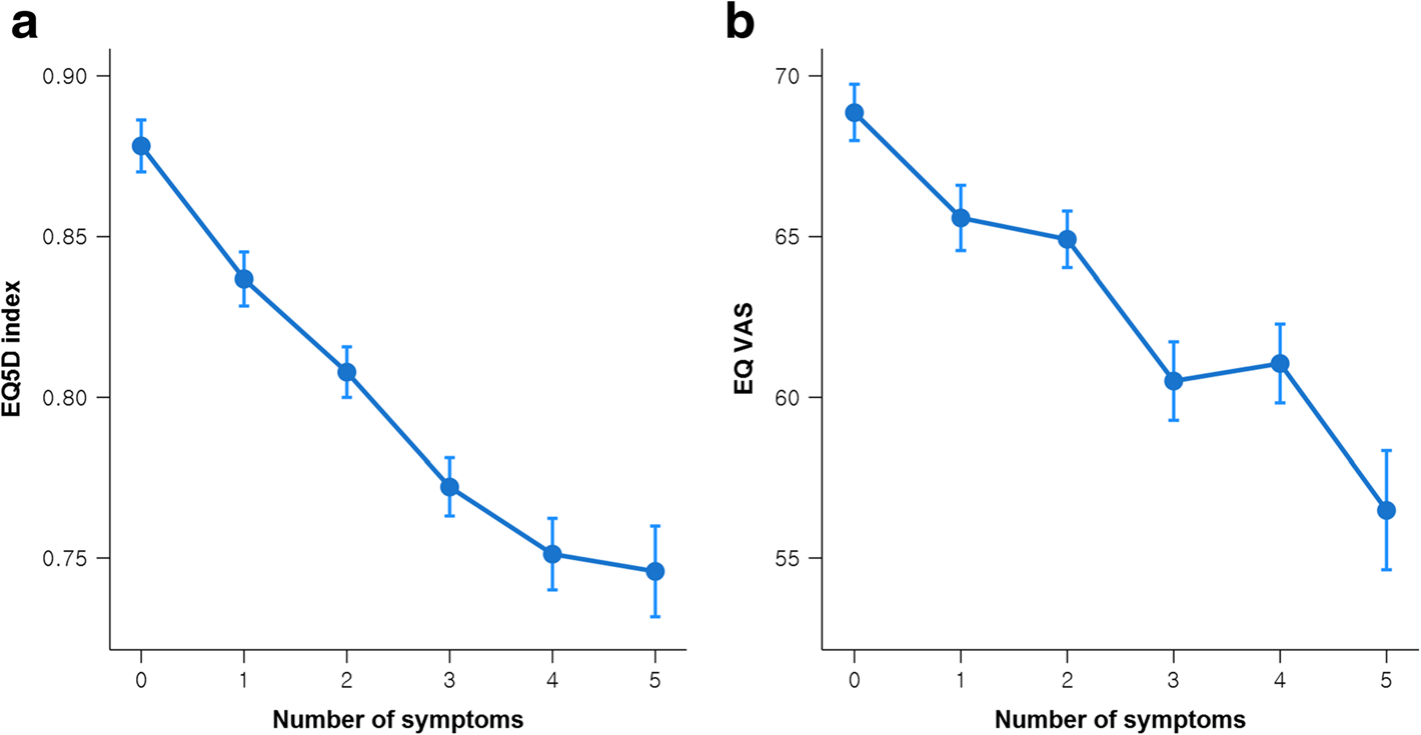
EQ-5D index scores (**a**) and EQ VAS scores (**b**) by number of symptoms

The EQ-5D index score was negatively correlated with the number of symptoms (Spearman’s correlation coefficient = −0.370, *p* < 0.01). The EQ VAS score was also significantly but more weakly correlated (Spearman’s correlation coefficient = −0.226, *p* < 0.01) (Fig. 2).

In the logistic regression analysis for each EQ-5D dimension, fatigue had the largest contribution to the mobility (adjusted OR = 2.179) and pain/discomfort dimensions (adjusted OR = 2.167), along with pain (adjusted OR for mobility = 2.996, adjusted OR for pain/discomfort = 3.106), and they had significant associations with all other dimensions, including self-care (fatigue: adjusted OR = 1.678, pain: adjusted OR = 1.643), usual activities (fatigue: adjusted OR = 1.762, pain: adjusted OR = 1.993), and anxiety/depression dimensions (fatigue: adjusted OR = 1.986, pain: adjusted OR = 1.498). Sleep disturbance was also significantly associated with all dimensions, with the highest OR for anxiety/depression (adjusted OR = 2.573). Indigestion was significantly associated with pain/discomfort (adjusted OR =1.864) and anxiety/depression dimensions (adjusted OR =1.502), while depression/anger/anxiety was significantly associated with usual activities (adjusted OR =1.340), pain/discomfort (adjusted OR =1.788), and anxiety/depression dimensions (adjusted OR =5.843) (Table 3).

**Table 3:** Estimated ORs of each symptom among the EQ-5D dimensions

		EQ-5D dimensions				
		Mobility	Self-care	Usual activities	Pain/discomfort	Anxiety/depression
Symptoms	Fatigue	2.179^**^ (1.680 to 2.827)	1.678^**^ (1.186 to 2.375)	1.762^**^ (1.366 to 2.271)	2.167^**^ (1.670 to 2.811)	1.986^**^ (1.533 to 2.571)
	Pain	2.996^**^ (2.301 to 3.902)	1.643^**^ (1.149 to 2.350)	1.993^**^ (1.538 to 2.583)	3.106^**^ (2.376 to 4.060)	1.498^**^ (1.154 to 1.944)
	Sleep disturbance	1.737^**^ (1.311 to 2.301)	1.596^**^ (1.142 to 2.231)	1.394^*^ (1.071 to 1.814)	1.585^**^ (1.187 to 2.118)	2.573^**^ (1.977 to 3.351)
	Indigestion	1.126 (0.835 to 1.517)	1.258 (0.872 to 1.813)	1.320 (0.993 to 1.754)	1.864^**^ (1.348 to 2.578)	1.502^**^ (1.134 to 1.988)
	Depression/anger/anxiety	1.187 (0.874 to 1.612)	1.376 (0.963 to 1.967)	1.340^*^ (1.007 to 1.784)	1.788^**^ (1.285 to 2.490)	5.843^**^ (4.309 to 7.923)

The probability of reporting moderate or extreme problems in the respective EQ-5D dimension was used as the dependent variable. All estimates were adjusted for gender, age, income, education, and solitary living. ^*^, *p* < 0.05; ^**^, *p* < 0.01

In the multiple regression analysis of the EQ-5D index, fatigue (*β* = −0.073, *p* < 0.05), pain (*β* = −0.140, *p* < 0.01), sleep disturbance (β = −0.061, *p* < 0.05), and depression/anger/anxiety (*β* = −0.065, *p* < 0.05) showed significant independent effects on the EQ-5D index, explaining 23.6 % of the variance in the EQ-5D index score (*p* < 0.01). In a regression model of the EQ VAS score, fatigue (*β* = −0.070, *p* < 0.05) and sleep disturbance (*β* = −0.063, *p* < 0.05) showed significant independent effects on the EQ VAS score, explaining 8.2 % of the variance in the EQ VAS score (*p* < 0.01) (Table 4).

**Table 4:** Multiple regression analysis of the EQ-5D index and EQ VAS scores

	EQ-5D index			EQ VAS score		
	B	SE	*β*	B	SE	*β*
Gender (reference: male)						
Female	−0.037	0.009	−0.127^**^	−0.806	1.159	−0.024
Age	−0.002	0.001	−0.077^*^	−0.066	0.089	−0.024
Education (reference: less than elementary school)						
Elementary school	0.034	0.009	0.123^**^	0.190	1.124	0.006
Middle school	0.042	0.013	0.106^**^	0.112	1.606	0.002
High school or higher	0.062	0.015	0.123^**^	−0.093	1.917	−0.002
Living alone (reference: no)						
Yes	0.003	0.010	0.010	3.055	1.205	0.082^*^
Household income (10,000 won/month) (reference: < 50)						
50-99	−0.008	0.010	−0.028	−1.085	1.286	−0.033
100-199	−0.010	0.011	−0.034	1.857	1.443	0.053
≥ 200	−0.002	0.014	−0.006	1.434	1.803	0.030
Fatigue	−0.020	0.008	−0.073^*^	−2.206	1.064	−0.070^*^
Pain	−0.039	0.009	−0.140^**^	−1.344	1.070	−0.042
Sleep disturbance	−0.018	0.008	−0.061^*^	−2.145	1.062	−0.063^*^
Indigestion	0.007	0.009	0.022	−0.705	1.111	−0.019
Depression/anger/anxiety	−0.020	0.009	−0.065^*^	−1.971	1.185	−0.054
Diagnosed diseases						
Arthritis	−0.043	0.008	−0.144^**^	−3.135	1.031	−0.090^**^
Osteoporosis	−0.025	0.010	−0.066^*^	−4.087	1.322	−0.092^**^
Parkinson’s disease	−0.104	0.046	−0.059^*^	−11.656	5.785	−0.057^*^
Asthma^a^ or Chronic hepatitis^b^	−0.035	0.016	−0.056^*^	−11.890	4.437	−0.076^**^
Dyslipidemia^a^ or Allergy^b^	−0.015	0.007	−0.055^*^	−4.955	2.136	−0.066^*^
Chronic gastritis	−0.022	0.010	−0.056^*^	−3.524	1.285	−0.079^**^
R^2^	0.249			0.098		
Adjusted R^2^	0.236			0.082		

*B* unstandardized coefficients, *SE* standard error of unstandardized coefficients, *β* standardized coefficients

^*^ *p* < 0.05; ^**^ *p* < 0.01, ^a^Selected using a forward stepwise selection in a regression model for the EQ-5D index

^b^Selected using a forward stepwise selection in a regression model for the EQ VAS

## Discussion

The present study shows that common symptoms experienced by elderly individuals—including fatigue, pain, sleep disturbance, indigestion, and depression/anger/anxiety—are correlated in the aging population of rural Korea. Of the participants who reported these symptoms, those who presented comorbid symptoms tended to report higher symptom severity than did those with only one symptom. Fatigue, pain, and sleep disturbance showed negative effects on all dimensions of EQ-5D. After adjusting for socioeconomic characteristics and diagnosed diseases, we found that the independent effects of fatigue, pain, sleep disturbance, and depression/anger/anxiety were significant for the EQ-5D index.

In general, HRQoL has been reported to decrease with age and to be lower in women than in men. HRQoL in rural areas is reported to be lower than that in urban areas. In this study, the same trends were observed for gender and age. Furthermore, the mean HRQoL of the assessed rural elderly population was lower than the mean HRQoL found in a nationwide sample of elderly individuals in Korea [18]. The mean EQ-5D index score in this study was relatively lower than the mean reported for older people in rural Vietnam using the same value set for calculating EQ-5D indices [19]. Compared with the reported prevalence of symptoms in non-elderly Korean adults, the prevalence of common symptoms in this study’s participants was generally higher, except for the prevalence of fatigue, which was lower in the elderly. These results are consistent with those of previous studies on elderly populations showing an increased prevalence of sleeping disturbance and pain and a decreased prevalence of general fatigue [20–22].

In this study, although each of the symptoms was positively correlated with the others, the strongest associations were observed between fatigue and pain and between fatigue and depression/anger/anxiety. Previous studies showed strong associations between these symptoms [4, 5], and fatigue, pain and depression have been commonly reported as a symptom cluster in patients with cancer [23] and multiple sclerosis [24]. Furthermore, individuals who reported certain comorbid symptoms tended to report higher symptom severity, and this trend was statistically significant for fatigue and pain. This result suggests that the presence of other symptoms not only affects the presence of fatigue and pain but also increases the perceived severity of fatigue and pain.

The common symptoms that showed significant independent effects on the EQ-5D index were fatigue, pain, sleep disturbance and depression/anger/anxiety, with pain having the largest effect. This result is similar to previous research reporting that pain was perceived as the most burdensome somatic symptom among older patients who visited family physicians [25]. Pain and fatigue have also been reported to contribute to the total burden of poor self-rated health in middle-aged and elderly people [26]. Moreover, fatigue, pain and sleep disturbance showed significantly negative effects on all dimensions of EQ-5D. Therefore, their effect on HRQoL is not limited to a specific dimension but encompasses a broad range of dimensions of HRQoL.

On the other hand, only fatigue and sleep disturbance showed independent effects on the EQ VAS, which is a direct patient-based instrument, in contrast to the EQ-5D index, which is an indirect community-based instrument [27]. The correlation between the EQ-5D index and the EQ VAS has been suggested to be associated with socio-demographic factors and medical conditions [28, 29]. Additionally, the EQ-5D includes domains with questions related to pain and depression. The different effects of the symptoms on the EQ-5D index and the EQ VAS score found in this study were likely a combination of these factors. Both the EQ-5D index and the EQ VAS consistently affected the number of symptoms, and this number was negatively correlated with HRQoL. This result is consistent with those of a study that demonstrated that the presentation of fewer symptoms was associated with self-rated health in community-living older individuals [30].

The common symptoms included in this study were mainly non-specific and difficult to interpret as resulting from any specific disease. The effects of the symptoms on HRQoL were significant even after we adjusted for disease status. Although healthcare strategies have primarily focused on treating patients with a specific disease or preventing a specific disease, multidisciplinary approaches based on the co-occurrence and interrelationships among common symptoms are also required to improve HRQoL in the aging population. For example, individual with one symptom should be screened for other symptoms that commonly tend to co-occur. Symptoms that have significant associations with HRQoL, i.e., fatigue, pain, sleep disturbance and depression/anger/anxiety, should be addressed with higher priority. Additionally, considering that fatigue and pain had the strongest association and contributed significantly to the mobility dimension of EQ-5D, increased mobility would be a good treatment goal for people with those symptoms, as it could lead to increased HRQoL.

Of the 5 common symptoms included in this study, only indigestion did not have an independent effect on the EQ-5D index. Gastrointestinal disorders such as functional dyspepsia have been reported to affect a patient’s quality of life [13, 31], but the severity of indigestion in the assessed population may have been too mild to affect their HRQoL. Additionally, although a number of gastrointestinal symptoms, such as upper abdominal pain or discomfort, bloating, belching, heartburn or diarrhea, affect elderly populations, a general expression (“problems with digestion”) was used in the questionnaire to keep the questionnaire concise and readily administered. Therefore, while this term was simple and easily understandable, it could be ambiguous in its precise definition, which may explain why the results of this study differ from those of previous studies. Thus, our findings related to indigestion should not be extended to apply to other gastrointestinal symptoms or disorders.

Several limitations of this study must be considered when interpreting the results. First, this study used data from a convenient sample of volunteers in the community. Although the sample was large and encompassed 22.4 % of the elderly population in the included towns [32], the study sample might not have been representative of the community and of the Korean elderly population. The proportion of females and elderly in their 70s in our sample was approximately 10 % higher than is observed in the general population of elderly people in Korea with a lower education level and higher obesity prevalence, while other socioeconomic characteristics and the prevalence of diagnosed diseases were similar. Second, a wider scope of human well-being that included social, environmental, and religious aspects was not comprehensively evaluated. Thus, the study results cannot be generalized to comprehensive quality of life and are limited to quality of life focusing on health. Third, the information on most diseases was self-reported, although self-reports of diseases have been shown to provide reasonable estimates in the older population [33]. Clinical parameters were also used for several prevalent diseases, including hypertension, diabetes, dyslipidemia and obesity. Fourth, we used binary variables for symptoms in the analysis of pairwise associations between symptoms and in the regression analysis of HRQoL. In future studies, we recommend using continuous variables that reflect the severity of symptoms to obtain additional insights. Finally, this study was cross-sectional, which did not allow for cause-effect interpretations of the associations between the reported symptoms and the participants’ quality of life. Thus, longitudinal studies should be conducted in the future to understand the causal relationship between such symptoms and quality of life.

## Conclusions

The present study showed that common symptoms experienced by the elderly, including fatigue, pain, sleep disturbance, indigestion, and depression/anger/anxiety, were correlated with one another in an older population of rural Korea. Moreover, fatigue, pain, sleep disturbance, and depression/anger/anxiety showed significant independent effects on HRQoL status after we adjusted for socioeconomic characteristics and diagnosed diseases. Longitudinal studies are required to determine the causal relationships between such symptoms and quality of life. Moreover, in addition to the current healthcare strategies used to target specific diseases, multidisciplinary approaches focusing on common symptoms are required to improve HRQoL in the elderly.

## Abbreviations

HRQoL: Health-related quality of life
OECD: The Organization for Economic Co-operation and Development
OR: Odds ratio
VAS: Visual analogue scale
